# EFFECT OF A HOME-BASED EXERCISE PROGRAM ON SUBSEQUENT FALLS IN SENIORS AFTER A FALL: A RANDOMIZED CLINICAL TRIAL

**DOI:** 10.1093/geroni/igz038.068

**Published:** 2019-11-08

**Authors:** Teresa Liu-Ambrose, Jennifer C Davis, John R Best, Larry Dian, Wendy Cook, Kenneth Madden, Chun Liang Hsu, Karim M Khan

**Affiliations:** 1 University of British Columbia, Vancouver, British Columbia, Canada; 2 University of British Columbia - Okanagan Campus, Kelowna, British Columbia, Canada; 3 Department of Medicine, Division of Geriatric Medicine, Faculty of Medicine, University of British Columbia, British Columbia, Canada; 4 Division of Geriatric Medicine, University of British Columbia, Vancouver, Canada; 5 Gerontology and Diabetes Laboratory, University of British Columbia, Vancouver, British Columbia, Canada; 7 Center for Hip Health and Mobility, Vancouver Coastal Health Research Institute, Vancouver, Canada

## Abstract

We assessed the efficacy of the home-based Otago Exercise Program (OEP) as a secondary falls prevention strategy in seniors referred to a falls prevention clinic after an index fall. We conducted a 12-month randomized controlled trial of 344 adults, aged 70 years and older, with = or > 1 fall resulting in medical attention in the prior 12 months. Participants were randomized to OEP or standard of care (CON). The OEP is a home-based strength and balance training program delivered by a physical therapist. All participants received AGS Guideline Care for falls prevention from a geriatrician. Differences in falls rate was tested with a negative binomial regression model. The rate of falls was lower in the OEP group vs the CON group (incident rate ratio [IRR] = 0.64, 95% CI 0.46 to 0.90). The estimated incidence rate of falls per person-year was 1.4 (95% CI 0.1 to 2.0) in the OEP group and 2.1 (95% CI 0.1 to 3.2) in the CON group, with an absolute incidence rate difference of 0.74 (95% CI 0.04 to 1.78) falls per person-year. DSST performance also increased in the OEP group by a mean change of 1.1 points (95% CI 0.02 to 2.1) vs the CON group. Improved DSST was associated with fewer falls (IRR = 0.80, 95% CI 0.68 to 0.95). These findings support the use of the OEP for secondary falls prevention.

